# 3-*exo*-Chloro-8-oxabicyclo­[3.2.1]oct-6-ene-2,4-diol chloro­form 0.33-solvate

**DOI:** 10.1107/S1600536809021898

**Published:** 2009-06-13

**Authors:** Viktor A. Tafeenko, Leonid A. Aslanov, Marina V. Proskurnina, Sergei E. Sosonyuk, Dmitrii A. Khlevin

**Affiliations:** a Chemistry Department, Moscow State University, 119991 Moscow, Russian Federation

## Abstract

The title compound, 3C_7_H_9_ClO_3_·CHCl_3_, crystallizes with mol­ecules of 3-*exo*-chloro-8-oxabicyclo­[3.2.1]oct-6-ene-2,4-diol (*A*) and chloro­form in a 3:1 ratio, in the space group *R*3*m*. Mol­ecules of *A* straddle a crystallographic mirror plane, whereas the chloro­form mol­ecules (C and H atoms) lie additionally on the threefold axis. The mol­ecules of *A* are linked into right- and left-helical chains by means of O—H⋯O hydrogen bonds, thus forming columns running along the *c* axis. Six inter­penetrated columns form a channel in which the solvent mol­ecules (chloro­form) are located.

## Related literature

Inositetriphosphates analogues are potential prospective anti­tumoral compounds, see: Piettre *et al.* (1997 ▶); Miller & Allemann (2007 ▶).
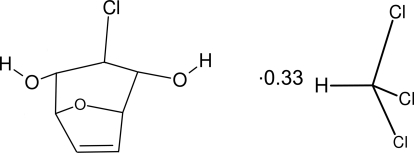


## Experimental

### 

#### Crystal data

3C_7_H_9_ClO_3_·CHCl_3_
*M* *_r_* = 649.16
Hexagonal, 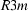

*a* = 18.687 (5) Å
*c* = 6.8723 (16) Å
*V* = 2078.3 (9) Å^3^
*Z* = 3
Cu *K*α radiation
μ = 6.09 mm^−1^
*T* = 295 K
0.1 × 0.07 × 0.06 mm

#### Data collection

Enraf–Nonius CAD-4 diffractometer
Absorption correction: ψ scan (North *et al.*, 1968 ▶) *T*_min_ = 0.530, *T*_max_ = 0.694
1832 measured reflections
987 independent reflections
966 reflections with *I* > 2σ(*I*)
*R*_int_ = 0.036
2 standard reflections frequency: 120 min intensity decay: none

#### Refinement

*R*[*F* ^2^ > 2σ(*F* ^2^)] = 0.054
*wR*(*F* ^2^) = 0.168
*S* = 1.18
987 reflections
69 parameters
1 restraint
H atoms treated by a mixture of independent and constrained refinement
Δρ_max_ = 0.28 e Å^−3^
Δρ_min_ = −0.51 e Å^−3^
Absolute structure: Flack (1983 ▶), 481 Friedel pairs
Flack parameter: −0.01 (2)

### 

Data collection: *CAD-4 Software* (Enraf–Nonius, 1989 ▶); cell refinement: *CAD-4 Software*; data reduction: *XCAD4* (Harms & Wocadlo, 1995 ▶); program(s) used to solve structure: *SHELXS97* (Sheldrick, 2008 ▶); program(s) used to refine structure: *SHELXL97* (Sheldrick, 2008 ▶); molecular graphics: *DIAMOND* (Brandenburg, 2000 ▶); software used to prepare material for publication: *WinGX* (Farrugia, 1999 ▶).

## Supplementary Material

Crystal structure: contains datablocks I, global. DOI: 10.1107/S1600536809021898/si2178sup1.cif

Structure factors: contains datablocks I. DOI: 10.1107/S1600536809021898/si2178Isup2.hkl

Additional supplementary materials:  crystallographic information; 3D view; checkCIF report

## Figures and Tables

**Table 1:** Hydrogen-bond geometry (Å, °)

*D*—H⋯*A*	*D*—H	H⋯*A*	*D*⋯*A*	*D*—H⋯*A*
O1—H11⋯O1^i^	0.77 (7)	1.98 (7)	2.723 (3)	162 (7)

Symmetry code: (i) 
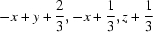
.

**Fig. 1.**
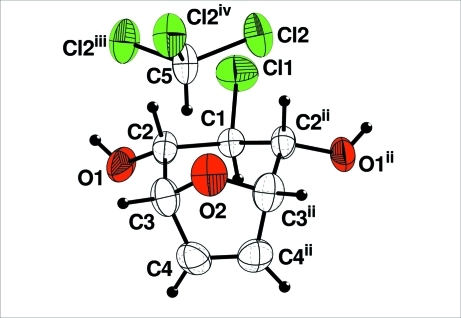
The molecular structure of the title compound, with displacement ellipsoids drawn at the 30% probability level. Symmetry codes: (ii) x, x-y, z; (iii) -y + 1, x-y, z; (iv) 1 - x + y, -x + 1, z.

**Fig. 2.**
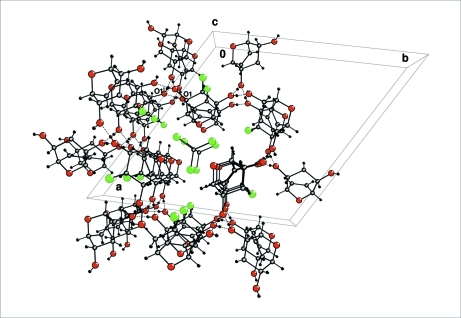
The packing motif of the crystal structure. Symmetry codes: (i) -x + y+2/3, -x + 1/3, z + 1/3

**Fig. 3.**
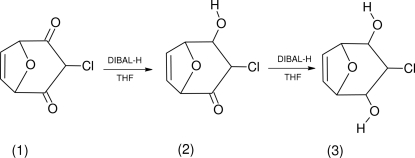
Reaction scheme.

## supplementary crystallographic information

### Comment

It is known (Piettre *et al.*, (1997); Miller & Allemann, (2007)) that the analogous of inositetriphosphates are potential prospective antitumoral compounds. As we assume that the polyhydroxycycloheptanes are able to act like inositemonophosphatase inhibitors, so the 3-*exo*-chloro-8-oxa-bicyclo[3.2.1]oct-6-en-2,4-diendo-diol was synthesized. The elemental analysis of the compound we obtained, were in good agreement with the title compound, but ^1^H NMR data did not allow us to clarify the relative oxy-groups arrangement in the molecule. To determine the structure of the compound, we carried out an X-ray crystallographic analysis. Molecule of (A) (Fig.1) straddle a crystallographic mirror plane m passing through atoms Cl1, C1, H1, endocyclic oxygen O2, the midpoint of the double bond C4/C4^ii^, whereas the chloroform molecules (carbon C5 and hydrogen H5 atoms) lie additionally on the threefold axis. The 6-membered cycle of the molecule adopts a chair conformation, with atoms O2 and C1 displaced out of plane defined by the atoms C2/C2^ii^/C3/C3^ii^ (plane 1) by -0.848 (3) and 0.543 (2) Å. Atoms O1, C4, Cl1, H1 (attached to C1) displaced out of plane 1 by 0.797 (2), 1.329 (3), 0.1990 (1), 1.44 (2) Å, while atoms H2 and H3 (attached to C2 and C3) by -.90 (3), -0.37 (3) Å, respectively. The packing motif, as shown in Fig.2 can be described as follows: the molecules (A) are linked into the interpenetrated right- and left-helical chains by means of O1—H···O1* hydrogen bonds thus to form columns, running along the *c* axis. The six interpenetrated columns form channels, where solvent molecules (chloroform) are located.

### Experimental

1*M* hexane solution of DIBAL-H (6 ml) was added (Fig. 3) dropwise during 20 min at 213 K to a solution of compound (1) 3-chloro-8-oxabicyclo[3.2.1]-6-en-2,4-dione (5.75 mmol, 1000 mg) in 40 ml of dry THF under argon atmosphere. Reaction quenched with methanol and the mixture stirred for 4–5 h, concentrated to 20–30 ml, filtered through silica gel (2 cm, 60/200 µ), evaporated to dryness to yield compound 2 as yellow oil which was used for preparing of compound (3) without further purification. 1*M* hexane solution of DIBAL-H (6 ml) was added dropwise during 20 min at 213 K to a solution of compound (2) (1.72 mmol, 300 mg) in 20 ml of dry THF under argon atmosphere. The reaction was quenched with methanol (15 ml) and the mixture was stirred for 4–5 h, concentrated to 10 ml, filtered through silica gel (2 cm, 60/200 µ), evaporated to dryness to yield compound (3) as a colorless solid (280 mg) which was flesh-chromatographed on silica gel (40/60 µ, CH_2_Cl_2_ Et_2_O: 2:1, gradient 1:5) to give diol (3) (150 mg), Crystals suitable for diffraction analysis were obtained by slow diffusion of petroleum ether vapour into a CHCl_3_ solution.

### Refinement

The positions of the H atoms were determined from Fourier difference maps; H atoms attached to carbons were then placed in calculated positions and allowed to ride on their parent atoms [C—H = 0.93–0.98 Å. *U*_iso_(H) = *xU*_eq_(parent atom), where *x* = 1.2.] Hydrogen attached to oxygen was refined freely.

### Crystal data

**Table:**

3C_7_H_9_ClO_3_·CHCl_3_	*D*_x_ = 1.556 Mg m^−^^3^
*M**_r_* = 649.16	Melting point: decomposition K
Hexagonal, *R*3*m*	Cu *K*α radiation, λ = 1.54184 Å
Hall symbol: R 3 -2"	Cell parameters from 25 reflections
*a* = 18.687 (5) Å	θ = 32–45°
*c* = 6.8723 (16) Å	µ = 6.09 mm^−^^1^
*V* = 2078.3 (9) Å^3^	*T* = 295 K
*Z* = 3	Prism, colourless
*F*(000) = 1002	0.1 × 0.07 × 0.06 mm

### Data collection

**Table:**

Enraf–Nonius CAD-4 diffractometer	966 reflections with *I* > 2σ(*I*)
Radiation source: fine-focus sealed tube	*R*_int_ = 0.036
graphite	θ_max_ = 71.9°, θ_min_ = 4.7°
Nonprofiled ω scans	*h* = −22→0
Absorption correction: ψ scan (North *et al.*, 1968)	*k* = 0→22
*T*_min_ = 0.530, *T*_max_ = 0.694	*l* = −8→8
1832 measured reflections	2 standard reflections every 120 min
987 independent reflections	intensity decay: none

### Refinement

**Table:**

Refinement on *F*^2^	Hydrogen site location: inferred from neighbouring sites
Least-squares matrix: full	H atoms treated by a mixture of independent and constrained refinement
*R*[*F*^2^ > 2σ(*F*^2^)] = 0.054	*w* = 1/[σ^2^(*F*_o_^2^) + (0.021*P*)^2^ + 2.2041*P*] where *P* = (*F*_o_^2^ + 2*F*_c_^2^)/3
*wR*(*F*^2^) = 0.168	(Δ/σ)_max_ = 0.001
*S* = 1.18	Δρ_max_ = 0.28 e Å^−^^3^
987 reflections	Δρ_min_ = −0.51 e Å^−^^3^
69 parameters	Extinction correction: *SHELXL97* (Sheldrick, 2008), Fc^*^=kFc[1+0.001xFc^2^λ^3^/sin(2θ)]^-1/4^
1 restraint	Extinction coefficient: 0.0016 (4)
Primary atom site location: structure-invariant direct methods	Absolute structure: Flack (1983), 481 Friedel pairs
Secondary atom site location: difference Fourier map	Flack parameter: −0.01 (2)

### Special details

**Table:**

Experimental. As the solvent molecules release from the crystal at ambient air, so the experiment was carried out from the crystal placed in a glass capillary.
Geometry. All esds (except the esd in the dihedral angle between two l.s. planes) are estimated using the full covariance matrix. The cell esds are taken into account individually in the estimation of esds in distances, angles and torsion angles; correlations between esds in cell parameters are only used when they are defined by crystal symmetry. An approximate (isotropic) treatment of cell esds is used for estimating esds involving l.s. planes.
Refinement. Refinement of *F*^2^ against ALL reflections. The weighted *R*-factor *wR* and goodness of fit *S* are based on *F*^2^, conventional *R*-factors *R* are based on *F*, with *F* set to zero for negative *F*^2^. The threshold expression of *F*^2^ > σ(*F*^2^) is used only for calculating *R*-factors(gt) *etc*. and is not relevant to the choice of reflections for refinement. *R*-factors based on *F*^2^ are statistically about twice as large as those based on *F*, and *R*- factors based on ALL data will be even larger.

### Fractional atomic coordinates and isotropic or equivalent isotropic displacement parameters (Å^2^)

**Table:**

	*x*	*y*	*z*	*U*_iso_*/*U*_eq_	
Cl1	0.29956 (13)	0.14978 (7)	0.6438 (3)	0.0671 (7)	
Cl2	0.61527 (5)	0.38473 (5)	0.7368 (3)	0.0619 (6)	
O1	0.3605 (3)	0.0525 (2)	0.3740 (6)	0.0560 (10)	
O2	0.5272 (3)	0.26359 (14)	0.3179 (9)	0.0583 (13)	
C1	0.3610 (3)	0.18052 (16)	0.4256 (8)	0.0341 (12)	
H1	0.3232	0.1616	0.3143	0.041*	
C2	0.4122 (3)	0.1378 (2)	0.4156 (6)	0.0397 (10)	
H2	0.4393	0.1436	0.5414	0.048*	
C3	0.4782 (3)	0.1795 (3)	0.2586 (8)	0.0510 (12)	
H3	0.5115	0.1527	0.2434	0.061*	
C4	0.4419 (4)	0.1856 (3)	0.0684 (8)	0.0543 (12)	
H4	0.4228	0.1467	−0.0311	0.065*	
C5	0.6667	0.3333	0.654 (2)	0.048 (3)	
H5	0.6667	0.3333	0.5111	0.058*	
H11	0.361 (4)	0.024 (4)	0.453 (10)	0.063 (19)*	

### Atomic displacement parameters (Å^2^)

**Table:**

	*U*^11^	*U*^22^	*U*^33^	*U*^12^	*U*^13^	*U*^23^
Cl1	0.0535 (11)	0.0777 (11)	0.0619 (10)	0.0268 (5)	0.0216 (8)	0.0108 (4)
Cl2	0.0497 (8)	0.0497 (8)	0.0932 (14)	0.0300 (8)	0.0052 (4)	−0.0052 (4)
O1	0.077 (3)	0.0298 (16)	0.058 (2)	0.0241 (18)	−0.0182 (19)	−0.0027 (15)
O2	0.026 (2)	0.054 (2)	0.085 (3)	0.0128 (11)	0.001 (2)	0.0003 (11)
C1	0.026 (3)	0.033 (2)	0.040 (3)	0.0132 (13)	−0.002 (2)	−0.0012 (11)
C2	0.039 (2)	0.0310 (19)	0.052 (2)	0.0196 (17)	−0.0126 (18)	−0.0054 (17)
C3	0.039 (2)	0.052 (3)	0.071 (3)	0.029 (2)	0.004 (2)	−0.003 (2)
C4	0.054 (3)	0.061 (3)	0.051 (2)	0.031 (2)	0.013 (2)	0.000 (2)
C5	0.033 (3)	0.033 (3)	0.078 (8)	0.0166 (16)	0.000	0.000

### Geometric parameters (Å, °)

**Table:**

Cl1—C1	1.799 (6)	C2—C3	1.526 (7)
Cl2—C5	1.759 (5)	C2—H2	0.9800
O1—C2	1.420 (5)	C3—C4	1.503 (8)
O1—H11	0.77 (7)	C3—H3	0.9800
O2—C3^i^	1.426 (6)	C4—C4^i^	1.320 (11)
O2—C3	1.426 (6)	C4—H4	0.9300
C1—C2	1.524 (5)	C5—Cl2^ii^	1.759 (5)
C1—C2^i^	1.524 (5)	C5—Cl2^iii^	1.759 (5)
C1—H1	0.9800	C5—H5	0.9800
			
C2—O1—H11	114 (5)	O2—C3—C2	105.7 (4)
C3^i^—O2—C3	102.6 (5)	C4—C3—C2	111.9 (4)
C2—C1—C2^i^	113.8 (5)	O2—C3—H3	111.8
C2—C1—Cl1	109.8 (3)	C4—C3—H3	111.8
C2^i^—C1—Cl1	109.8 (3)	C2—C3—H3	111.8
C2—C1—H1	107.7	C4^i^—C4—C3	107.6 (3)
C2^i^—C1—H1	107.7	C4^i^—C4—H4	126.2
Cl1—C1—H1	107.7	C3—C4—H4	126.2
O1—C2—C1	110.1 (4)	Cl2^ii^—C5—Cl2^iii^	110.0 (4)
O1—C2—C3	110.7 (4)	Cl2^ii^—C5—Cl2	110.0 (4)
C1—C2—C3	108.8 (4)	Cl2^iii^—C5—Cl2	110.0 (4)
O1—C2—H2	109.1	Cl2^ii^—C5—H5	108.9
C1—C2—H2	109.1	Cl2^iii^—C5—H5	108.9
C3—C2—H2	109.1	Cl2—C5—H5	108.9
O2—C3—C4	103.3 (4)		

Symmetry codes: (i) *x*, *x*−*y*, *z*; (ii) −*x*+*y*+1, −*x*+1, *z*; (iii) −*y*+1, *x*−*y*, *z*.

### Hydrogen-bond geometry (Å, °)

**Table:**

*D*—H···*A*	*D*—H	H···*A*	*D*···*A*	*D*—H···*A*
O1—H11···O1^iv^	0.77 (7)	1.98 (7)	2.723 (3)	162 (7)

Symmetry codes: (iv) −*x*+*y*+2/3, −*x*+1/3, *z*+1/3.
